# Evaluation of ^68^Ga-PSMA-11 PET/CT: a Phase 1 clinical study in Japanese patients with primary, recurrent, or suspected recurrent prostate cancer

**DOI:** 10.1007/s12149-024-01931-7

**Published:** 2024-05-16

**Authors:** Anri Inaki, Atsushi Mizokami, Hiroshi Wakabayashi, Kouji Izumi, Yoshifumi Kadono, Tadashi Toyama, Shizuko Takahara, Toshinori Murayama, Seigo Kinuya

**Affiliations:** 1 Department of Nuclear Medicine, Kanazawa University Hospital, 13-1 Takara-Machi, Kanazawa-Shi, Ishikawa, 920-8641 Japan; 2 Department of Integrative Cancer Therapy and Urology, Kanazawa University Hospital, 13-1 Takara-Machi, Kanazawa-Shi, Ishikawa, 920-8641 Japan; 3 Innovative Clinical Research Center, Kanazawa University Hospital, 13-1 Takara-Machi, Kanazawa-Shi, Ishikawa, 920-8641 Japan; 4 Department of Clinical Development, Kanazawa University Hospital, 13-1 Takara-Machi, Kanazawa-Shi, Ishikawa, 920-8641 Japan

**Keywords:** Prostate cancer, Positron emission imaging (PET), Prostate-specific membrane antigen (PSMA), PSMA PET, ^68^Ga-PSMA-11

## Abstract

**Background:**

Prostate-specific membrane antigen (PSMA)-targeted radiopharmaceuticals allow whole-body imaging to detect prostate cancer (PC). Positron emission tomography imaging using gallium-68 (^68^Ga)-PSMA-11 has been shown to have a favorable safety and tolerability profile and high diagnostic performance. The study evaluates the safety and pharmacokinetics of ^68^Ga-PSMA-11 in Japanese patients with primary, recurrent, or suspected recurrent prostate cancer.

**Methods:**

This single arm study enrolled Japanese patients with primary PC (*n* = 3), suspected recurrent PC following radical prostatectomy (*n* = 4), or suspected recurrent PC following radical radiotherapy (*n* = 3). All patients received a single intravenous dose of ^68^Ga-PSMA-11 2.0 MBq/kg (±10%) followed by PSMA PET imaging and safety and pharmacokinetic evaluations. Based on the blood concentrations of ^68^Ga-PSMA-11 and the radioactivity distribution rate in each organ/tissue, the absorbed doses in major organs/tissues and the whole-body effective dose were calculated by the Medical Internal Radiation Dose method.

**Results:**

Ten patients were enrolled. Mean age was 73.3 ± 4.8 years, and median prostate-specific antigen was 8.250 ng/mL. Five patients (50%) experienced a total of 6 adverse events, and no grade ≥ 2 adverse events or serious adverse events were reported. No clinically significant changes in vital signs, haematology parameters, or blood chemistry or ECG abnormalities were observed. The estimated whole body effective dose of ^68^Ga-PSMA-11 (mean ± standard deviation) was 2.524 × 10^–2^ ± 2.546 × 10^–3^ mSv/MBq. Time to maximum concentration (1.16 × 10^–4^ ± 1.3 × 10^–5^% ID/mL) in whole blood was 2.15 ± 0.33 min.

**Conclusions:**

^68^Ga-PSMA-11 has a favourable safety and tolerability profile in Japanese patients with primary, recurrent, or suspected recurrent prostate cancer, which is comparable to previous observations in other populations.

## Introduction

Prostate cancer (PC) has increasing significance: it is the second most frequent cancer diagnosis in patients and the fifth leading cause of cancer death worldwide [1]. In Japan, the incidence of PC has been growing rapidly over the past decades. The age-standardized incidence rate rose from 7.1 in 1975 to 58.7 per 100,000 in 2014 [2]. PC remains the sixth major cause of death in Japanese patients [2] and was the leading cancer-related cause of death in 2020 [3].

Imaging of the prostate-specific membrane antigen (PSMA) has become an important tool for managing patients with PC, and PSMA positron emission imaging (PET) is now part of the diagnostic flowchart for PC in international guidelines [4]. In primary PC, PSMA PET has shown superiority to cross-sectional imaging for the detection of pelvic lymph nodes and distant metastases. In PC recurrence, higher detection rates have been observed for PSMA PET than for any other available imaging techniques, especially at low prostate-specific antigen (PSA) values [4].

One of the most frequently employed radiotracers is gallium-68 (^68^Ga)-PSMA-11 [5], which is currently approved for use in the United States, Australia, and Canada. In the multicentre proPSMA study, 302 patients with untreated, biopsy-proven PC were randomly assigned to undergo ^68^Ga-PSMA-11 PET/computerized tomography (CT) or conventional imaging for the evaluation of pelvic nodal and distant metastatic disease [6]. Accuracy was 27% (95% confidence interval [CI], 23%–31%; *p* < 0.0001) greater with ^68^Ga-PSMA-11 PET/CT than with conventional imaging (92% vs. 65%, respectively). Sensitivity and specificity were also greater with ^68^Ga-PSMA-11 PET/CT (85% and 98%, respectively) compared to conventional imaging (38% and 91%, respectively) [6]. In a single arm study of 764 patients with histopathology-proven PC in the United States, sensitivity and specificity of ^68^Ga-PSMA-11 PET for primary staging was 40% and 95%, respectively [7]. In a prospective study that enrolled 635 patients with biochemically recurrent PC, ^68^Ga-PSMA-11 imaging had a positive predictive rate of 92% and detection rate of 75% [8].

We aimed to evaluate the safety of ^68^Ga-PSMA-11, administered using the injection kit for PET/CT imaging in a sample of Japanese patients with primary, recurrent, or suspected recurrent PC.

## Materials and methods

### Study design

This was a single-centre, open-label, prospective phase I study approved by the Institutional Review Board of Kanazawa University Hospital and registered with the Japanese Registry of Clinical Trials (identifier: JRCT2041200110). The study was conducted in accordance with the ethical standards of the Declaration of Helsinki and International Council for Harmonisation of Technical Requirements for Pharmaceuticals for Human Use Guideline for Good Clinical Practice. All patients provided written informed consent for participation in the study. The objectives of the study were to evaluate the safety and pharmacokinetics of ^68^Ga-PSMA-11 used for PET/CT scans in patients with primary, recurrent, or suspected recurrent PC.

### Patient population

**Fig. 1:**
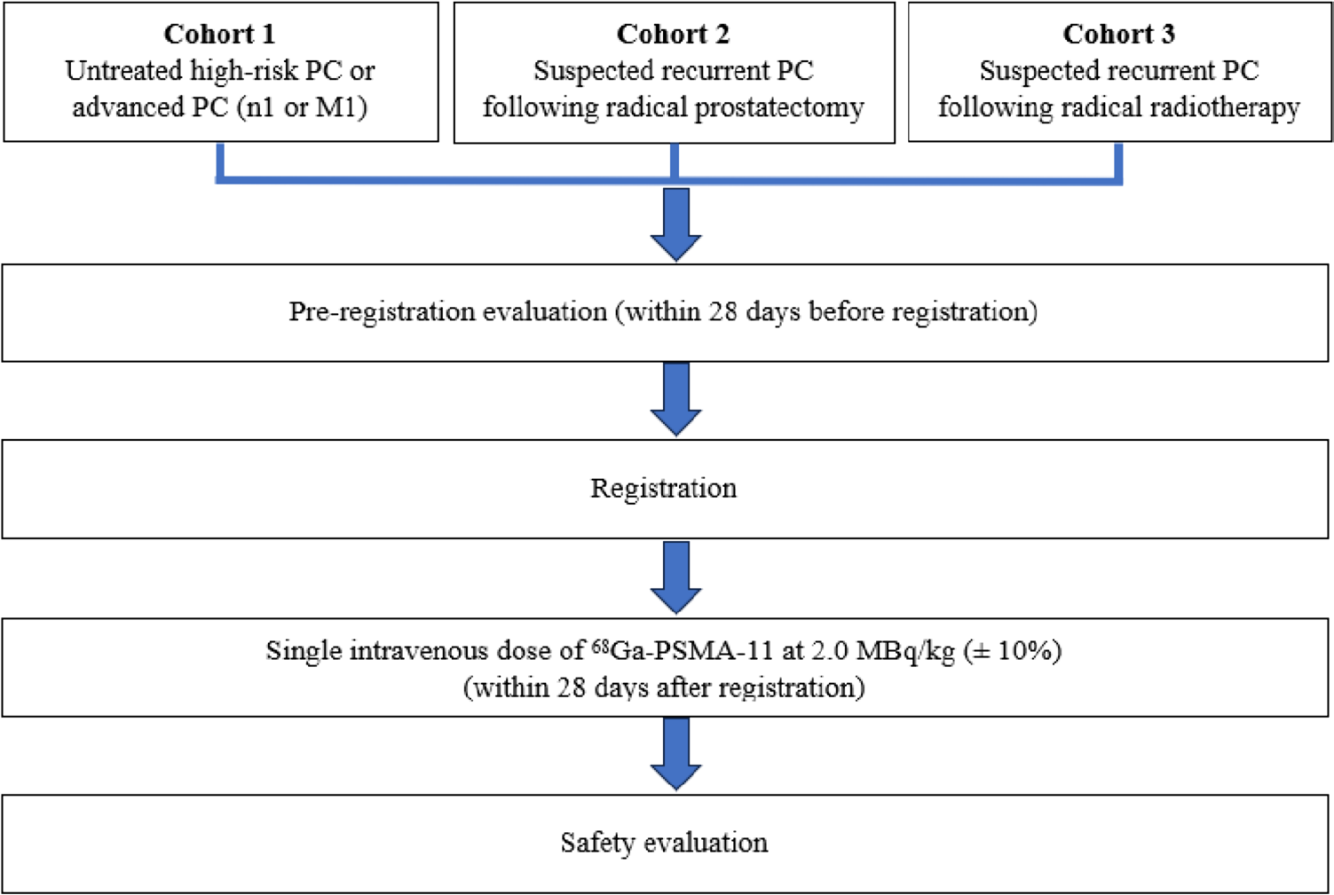
Study diagram

Japanese males (> 20 years of age) with primary, recurrent, or suspected recurrent PC were divided into 3 investigative cohorts (Fig. 1). Cohort 1 included patients with histologically confirmed untreated high-risk PC or advanced PC (N1 or M1). Cohort 2 included patients with histologically confirmed PC, treated with radical prostatectomy at initial onset, who then had recurrent disease in a local or metastatic lesion and an increase in blood PSA concentration from baseline and suspected recurrence on imaging (such as bone scintigraphy, CT, magnetic resonance imaging (MRI), and fluorodeoxyglucose-PET). Cohort 3 included patients with histologically confirmed PC at the initial onset, treated with radical radiotherapy (external irradiation, interstitial irradiation, or its combination) who then had confirmed recurrent PC in a locally recurrent or metastatic lesion, an increase in blood PSA concentration from baseline and suspected recurrence on imaging. All patients were required to have Eastern Cooperative Oncology Group (ECOG) performance status (PS) of 0 or 1 within 28 days prior to study enrolment and agree to use proper contraception until 90 days after investigational drug administration. Patients with an active second cancer, virus infections or other infections or conditions necessitating treatment during the study period or with previous exposure to ^68^Ga-PSMA-11, were excluded from this study.

### ^68^Ga-PSMA-11 administration

A single intravenous dose of ^68^Ga-PSMA-11 2.0 MBq/kg (± 10%) was administered using the ^68^Ga-PSMA-11 injection kit provided by Telix Pharmaceuticals and ^68^Ge/^68^Ga generator AX001 (Fig. 1). Briefly, gallium (^68^Ga) chloride and 25 µg of PSMA-11 reconstituted with acetate buffer were transferred into a sterile vacuumed vial. Quality control was performed according to the manufacturer’s recommendations by visual examination, pH testing, and instant thin layer chromatography. ^68^Ga-PSMA-11 was administered within 28 days of trial registration.

### PSMA PET imaging

PET/CT imaging was performed using a Philips Vereos PET-CT system (serial no: 9000105). The imaging protocol was designed to capture a comprehensive head-to-toe assessment, spanning approximately 17–18 bed positions per patient. Patients were scanned in each bed position for 180 s. The temporal acquisition points were set at 5, 60, and 120 min following administration of ^68^Ga-PSMA-11.

Image reconstruction parameters were optimized to ensure image quality and quantitative accuracy. A Pool Phantom was used to encapsulate ^68^Ga, and then the uniformity of the radiotracer distribution and a standardized uptake value of 1 were validated. The reconstruction algorithm utilized was Ordered Subset Expectation Maximization combined with Time-of-Flight information. The specific settings for the reconstruction were as follows: 2 iterations, 11 subsets, and a 3 mm Gaussian filter. Attenuation correction was performed using CT-based attenuation correction, while scatter correction was achieved through the Single Scatter Simulation method. The reconstructed images had a voxel size of 2 × 2x2 mm in the X, Y, and Z dimensions. The field of view for PET imaging was set to 567 mm and for CT-AC was set to 600 mm.

### Safety assessment

Safety and tolerability assessment was based on physical examination, vital signs, haematology, serum chemistry, urinalysis, 12-lead electrocardiograms, MRI, CT, PSMA PET together with subjective findings and ECOG PS at predefined time intervals. Adverse events were defined as any untoward medical occurrence (including an abnormal laboratory value), whether or not related to the investigational drug. Adverse reactions were defined as adverse events for which a causal relationship with the investigational drug could not be ruled out. All adverse events and adverse reactions were graded according to Common Terminology Criteria for Adverse Events (CTCAE version 5).

Adverse events were collected from drug administration through Day 7 follow-up. Vital signs and subjective and objective symptoms were assessed prior to study entry and at 30, 60, 120 min and 7 days after administration of ^68^Ga-PSMA-11. Laboratory tests were performed on venous blood samples collected prior to dosing and 120 min and 7 days after administration.

### Pharmacokinetics

Following ^68^Ga-PSMA-11 administration, blood samples were collected at 2, 5, 30, 60, and 120 min for pharmacokinetic and blood radioactivity evaluation. Urine samples for pharmacokinetic and radioactivity evaluation were collected at 60 and 120 min post-administration, and the well-counter Hitachi-Aloka DCM-200 was used to analyze blood and urine samples.

### Dosimetry

The effective dose (mSv/MBq) in the whole body and the absorbed dose (mGy/MBq) in each organ were evaluated. The effective dose in the whole body was calculated based on the estimated absorbed doses of ^68^Ga-PSMA-11 in major organs/tissues using tissue weighting factors as described in the International Commission on Radiological Protection Publication 103 using the Medical Internal Radiation Dose method [9]. Phoenix Winnonlin was used to calculate dosimetry, and OLINDA2 was used to calculate absorbed and effective doses.

### Statistical analysis

Pharmacokinetic parameters, including area under the blood concentration–time curve (AUC), clearance (CL), elimination phase volume of distribution (V_z_), and elimination half-life (T^1^/_2z_) were calculated. Grade ≥ 3 safety events (and ≥ Grade 2 for adverse events other than laboratory values) were summarised using descriptive statistics. Absorbed and effective dose estimates were calculated by the medical internal radiation dose method and means, medians, and standard deviations (SD) are reported.

## Results

**Fig. 2:**
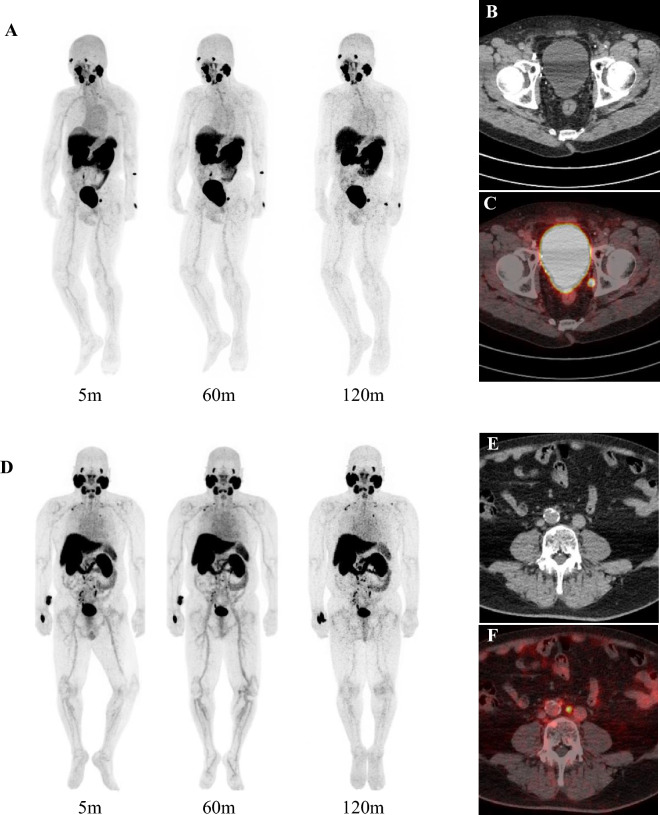
Representative ^68^Ga-PSMA-11 PET/CT images. **A** PET maximum intensity projection at 5, 60, and 120 min after injection, **B** axial CT, and **C** axial ^68^Ga-PSMA-11 PET/CT of a 74-year-old patient with initial PSA of 6.2 ng/mL and Gleason score 3 + 5. **D** PET maximum intensity projection at 5, 60, and 120 min after injection, **E** axial CT, **F** axial ^68^Ga-PSMA-11 PET/CT of a 70-year-old patient with an initial PSA of 5.96 ng/mL and Gleason score 3 + 4

In total, ten male patients were included (*n* = 3, *n* = 4, and *n* = 3 in Cohorts 1, 2, and 3, respectively). Cohort 1 included patients with untreated high-risk PC or advanced PC (N1 or M1). Cohort 2 included patients with suspected recurrent PC following radical prostatectomy. Cohort 3 included patients with suspected recurrent PC following radical radiotherapy (external irradiation, interstitial irradiation, or its combination). All patients underwent dosing with ^68^Ga-PSMA-11 followed by PET imaging. Representative PET/CT images are depicted in Fig. 2.

Baseline demographics are presented in Table 1. The mean age was 73.3 years, all patients had ECOG performance status of 0. PSA (Mean ± SD) was 33.211 ± 70.692 ng/mL in all patients, 92.720 ± 120.943 ng/mL in Cohort 1, 1.003 ± 1.181 ng/mL in Cohort 2, and 16.647 ± 7.762 ng/mL in Cohort 3.

**Table 1:** Demographics and baseline characteristics by patient

Patient #	Cohort	Age (years)	Body weight (kg)	Height (cm)	PSA (ng/mL)	Gleason score	TNM classification at enrollment
1	2	74	69.5	165.0	0.799	4 + 5	TxN0M0
2	1	69	66.4	178.3	40.500	4 + 4	T3aN0M0
3	3	84	62.5	163.3	9.840	5 + 4	T0N0M1b
4	2	73	66.4	168.4	0.227	3 + 3	TxN0M1a
5	2	70	57.0	166.4	0.257	4 + 4	TxN0M0
6	3	89	75.7	165.3	15.000	4 + 3	T0N0M0
7	1	71	69.6	167.5	231.000	4 + 4	T3bN1M1a
8	1	71	75.5	161.3	6.660	4 + 3	TxN0M0
9	2	73	73.2	174.0	2.730	4 + 3	TxN1M0
10	3	69	91.3	177.0	25.100	3 + 3	T0N0M1b

*PSA* prostate-specific antigen, *TNM* tumour, lymph nodes, metastasis Union for International Cancer Control staging system, *T0* no local PCa lesion due to hormone therapy or radiation therapy, *Tx* no prostate due to radical prostatectomy

### Safety

Five patients (50%) experienced a total of 6 adverse events: 66.7% (2/3 patients) in Cohort 1, 50.0% (2/4 patients) in Cohort 2, and 33.3% (1/3 patients) in Cohort 3 (Table 2). These included one occurrence each of injection site haemorrhage, injection site pain, injection site discomfort, amylase increased, neck pain, and dysuria. No Grade ≥ 2 adverse events or serious adverse events were observed in any patient. The events of injection site pain, injection site discomfort, and dysuria were also considered to be adverse reactions (adverse events that may be related to experimental treatment). One patient each reported increased amylase, neck pain, and dysuria. The dysuria event was determined to be related to ^68^Ga-PSMA-11, while amylase and neck pain events were determined to not be related. Adverse reactions were reported by 30.0% (3/10 patients) overall, 33.3% (1/3 patients) in Cohort 1, 25.0% (1/4 patients) in Cohort 2, and 33.3% (1/3 patients) in Cohort 3.

**Table 2:** Grade 1 adverse events in all cohorts

	All patients (*n* = 10)	Cohort 1 (*n* = 3)	Cohort 2 (*n* = 4)	Cohort 3 (*n* = 3)
Patients with adverse events, *n* (%)	5 (50.0)	2 (66.7)	2 (50)	1 (33.3)
Reported adverse events, *n*	6	2	3	1
General disorders and administration site conditions, *n* (%)	3 (30.0)	0	2 (50)	1 (33.3)
Injection site haemorrhage, *n* (%)	1 (10)	0	1 (25)	0
Injection site pain, *n* (%)	1 (10)	0	0	1 (33.3)
Injection site discomfort, *n* (%)	1 (10)	0	1 (25)	0
Investigations, *n* (%)	1 (10)	0	1 (25)	0
Amylase increased, *n* (%)	1 (10)	0	1 (25)	0
Musculoskeletal and connective tissue disorders, *n* (%)	1 (10)	1 (33.3)	0	0
Neck pain, *n* (%)	1 (10)	1 (33.3)	0	0
Renal and urinary disorders, *n* (%)	1 (10)	1 (33.3)	0	0
Dysuria, *n* (%)	1 (10)	1 (33.3)	0	0

No Grade ≥ 2 adverse events or serious adverse events were observed in any patient

No clinically significant changes in vital signs, haematology parameters, or blood chemistry, were observed throughout the study. No electrocardiogram abnormalities were observed for any patient.

### Whole-body distribution and dose estimation

Estimated absorbed doses in major organs/tissues are given in Table 3. The estimated whole body effective dose (mean ± SD) was 2.524 × 10^–2^ ± 2.546 × 10^–3^ mSv/MBq.

**Table 3:** Estimated absorbed doses of ^68^Ga-PSMA-11 in major organs

Target Organ (*n* = 10)	Estimated absorbed dose of ^68^Ga-PSMA-11, mGy/MBq	
	Median	Mean ± SD
Adrenals	4.035 × 10^–2^	4.109 × 10^–2^ ± 1.125 × 10^–2^
Brain	2.150 × 10^–3^	2.545 × 10^–3^ ± 1.594 × 10^–3^
Oesophagus	9.150 × 10^–3^	9.180 × 10^–3^ ± 5.187 × 10^–4^
Eyes	6.285 × 10^–3^	6.303 × 10^–3^ ± 5.397 × 10^–4^
Gallbladder wall	1.560 × 10^–2^	4.632 × 10^–2^ ± 9.111 × 10^–2^
Left colon	1.780 × 10^–2^	2.101 × 10^–2^ ± 1.271 × 10^–2^
Small intestine	3.865 × 10^–2^	3.893 × 10^–2^ ± 8.457 × 10^–3^
Stomach wall	1.420 × 10^–2^	1.516 × 10^–2^ ± 2.746 × 10^–3^
Right colon	1.475 × 10^–2^	1.467 × 10^–2^ ± 2.312 × 10^–3^
Rectum	1.085 × 10^–2^	1.095 × 10^–2^ ± 1.028 × 10^–3^
Heart wall	2.125 × 10^–2^	2.308 × 10^–2^ ± 6.016 × 10^–3^
Kidneys	0.3405	0.3171 ± 0.1178
Liver	3.215 × 10^–2^	3.30.6 × 10^–2^ ± 6.040 × 10^–3^
Lungs	2.045 × 10^–2^	2.117 × 10^–2^ ± 4.202 × 10^–3^
Pancreas	1.550 × 10^–2^	1.645 × 10^–2^ ± 3.131 × 10^–3^
Prostate	1.185 × 10^–2^	1.207 × 10^–2^ ± 1.537 × 10^–3^
Salivary glands	0.1180	0.1201 ± 3.944 × 10^–2^
Red marrow	9.840 × 10^–3^	1.091 × 10^–2^ ± 2.623 × 10^–3^
Osteogenic cells	1.002 × 10^–2^	1.211 × 10^–2^ ± 4.755 × 10^–3^
Spleen	4.015 × 10^–2^	4.403 × 10^–2^ ± 1.883 × 10^–2^
Testes	9.825 × 10^–3^	1.074 × 10^–2^ ± 4.120 × 10^–3^
Thymus	7.865 × 10^–3^	9.271 × 10^–3^ ± 3.458 × 10^–3^
Thyroid	8.615 × 10^–3^	9.979 × 10^–3^ ± 6.332 × 10^–3^
urinary bladder wall	0.1410	0.1497 ± 7.394 × 10^–2^
Total body	1.160 × 10^–2^	1.167 × 10^–2^ ± 2.584 × 10^–4^

*MBq* megabecquerel, *mGy* milli-gray, *SD* standard deviation

### Pharmacokinetics

**Fig. 3:**
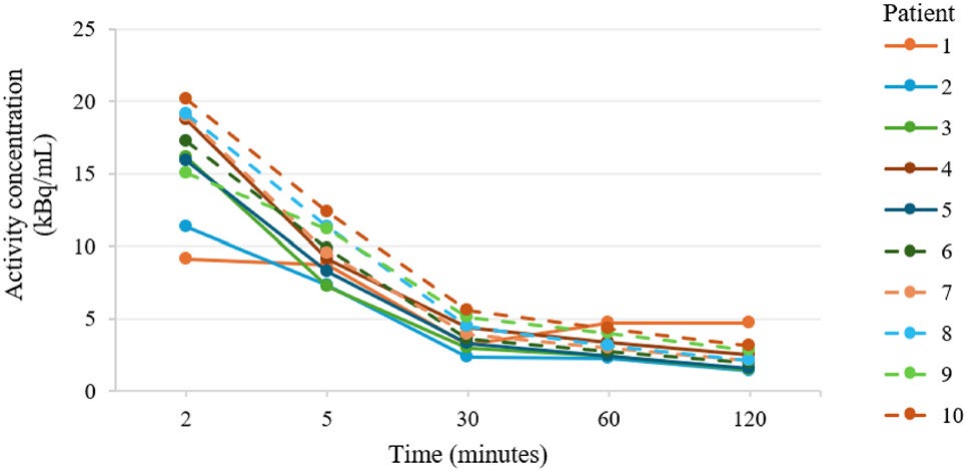
Time activity curve of ^68^Ga-PSMA-11 blood concentration by patient at 2, 5, 30, 60, and 120 min after injection

Blood concentrations for individual patients from 2 to 120 min after ^68^Ga-PSMA-11 administration are shown in Fig. 3. Time to maximum concentration (mean ± SD: 1.16 × 10^–4^ ± 1.3 × 10^–5^% ID/mL) in whole blood was 2.15 ± 0.33 min. Individual blood concentrations ranged from 9.16 to 20.16 kBq/mL at 2 min and decreased to 1.38 to 4.69 kBq/mL by 120 min.

Pharmacokinetic parameters (mean ± SD, *n* = 9) were as follows: T^1^/_2z_, 94.16 ± 9.04 min; AUC from dose administration to last measurable concentration (AUC_last_), 2.828 × 10^–3^ ± 3.18 × 10^–4^ min × % ID/mL; AUC from dose administration until elimination from the body (AUC_inf_), 4.803 × 10^–3^ ± 7.77 × 10^–4^ min × %ID/mL; Vz, 28.64268 ± 3.15303 L; and CL, 213.01 ± 33.67 mL/minute.

## Discussion

Accurate diagnostic imaging is crucial for detecting local recurrence or metastatic lesions in patients with prostate cancer [10]. Conventional imaging, such as CT, MRI, or bone scintigraphy, has been historically used to determine the extent of lymph node involvement and presence of metastases [10]. However, conventional imaging is limited in specificity and positivity [11–15], potentially subjecting patients to unnecessary harmful and costly procedures [16]. PSMA-targeted radiopharmaceuticals allow whole-body imaging for the detection of PC spread, and their increased sensitivity and specificity make them ideally suited for front-line imaging [17]. They are also associated with higher detection rates compared with other agents used in PC imaging [18]. Globally, PSMA PET is an emerging standard of care in PC imaging [4, 6, 19] with several products either approved or in development worldwide.

^68^Ga-PSMA-11 has been used in clinical practice outside Japan for approximately four years and the updated Society of Nuclear Medicine & Molecular Imaging Appropriate Use Criteria include ^68^Ga-PSMA-11 PET/CT as an alternative to standard bone and soft tissue imaging [20]. Since its development in 2012 [21], the ^68^Ga-PSMA-11 injection kit has been widely used in PET/CT imaging of prostate cancer and evaluated in many clinical studies, and a vast amount of clinical data have been obtained worldwide in prospective studies, retrospective studies, the phase 3 VISION study, and investigator-initiated clinical trials [7, 8, 22–27]. Due to the widely available clinical data already available in healthy volunteers, this study sought to confirm the favourable safety and tolerability of ^68^Ga-PSMA-11 in the Japanese patient population compared to other populations [6, 7]. As in studies in other populations, no serious adverse events were observed in any cohorts in this trial.

A purification step is not required for ^68^Ga-PSMA-11, unlike other radiolabelling methods for PSMA, enabling rapid radiolabelling at room temperature with high radiochemical purity and production consistency, suited to the hospital radio-pharmacy setting. A commercially available ^68^Ga-based PSMA PET imaging kit has been approved in Canada, New Zealand, and Australia. In the United States, the Food and Drug Administration has approved for patients with suspected metastasis who are candidates for initial definitive therapy, patients with suspected recurrence based on elevated serum prostate-specific antigen (PSA) level, and for selection of patients with metastatic prostate cancer, for whom lutetium-177 (^177^Lu) vipivotide tetraxetan PSMA-directed therapy is indicated [28].

This trial also confirmed the blood concentrations of ^68^Ga-PSMA-11 and its pharmacokinetic parameters, as well as the estimated exposure dose of ^68^Ga-PSMA-11 in the organs/tissues and whole body in Japanese men. In addition, the time-course of blood drug concentration and the distribution of radioactivity in the Japanese population observed in this clinical trial were similar to those in the non-Japanese population observed in overseas clinical trials [6, 7].

Recently, results from two phase 1/2 trials exploring ^18^F-PSMA-1007 PET/CT imaging for Japanese patients with prostate cancer have been published [29, 30]. Direct comparison of radiotracer performance is challenging and has not been studied in a large, prospective trial with lesion validation [31]. However, of available radiotracers, ^68^Ga-PSMA-11 has the most real-world evidence and studied most in clinical trials [31]. Interpretation of bone lesions, largely due to unspecific uptake, is more difficult on imaging acquired with ^18^F-labelled radiotracers than with ^68^Ga-PSMA-11 [32–35]. ^18^F-PSMA-1007 imaging has been shown to have significantly higher rates of nonspecific uptake, or equivocal bone lesions, than ^68^Ga-PSMA-11 imaging [36].

A recent comprehensive review has suggested that, from an imaging point of view, ^68^Ga-PSMA-11 PET/CT is unquestionably one of the most useful tools for the therapeutic management of patients with PC in the clinical setting in 2020 and foreseeable future [5]. Our trial results demonstrate that ^68^Ga-PSMA-11 has a favourable safety and tolerability profile for Japanese patients.

## Conclusion

^68^Ga-PSMA-11 has a favourable safety and tolerability profile in Japanese patients with primary, recurrent, or suspected recurrent prostate cancer. The time-course of blood drug concentration and the distribution of radioactivity in the Japanese population are comparable to previous observations in other populations.

## Data Availability

The datasets used and/or analyzed during the current study are available for the corresponding author on reasonable request.
